# Supplementary material for: Identifying the predisposing factors, signs and symptoms of overreaching and overtraining in physical education professionals
Source: PeerJ. 2018 Jun 13;6:e4994. doi: 10.7717/peerj.4994 (PMC6004110; doi:10.7717/peerj.4994)
Supplement: Supplemental Information 1 [file peerj-06-4994-s001.pdf]

Universidade Federal de Goiás
Faculdade de Educação Física e Dança
Setor de Fisiologia Humana e do Exercício

GENERAL DATA

Name:____
Date of birth: ___/_______/_____
Body mass: ______ **kg**
Height: ______ **cm**
Sex: ☐ **Male** ☐ **Female**
Training session duration (hours) _______
Weekly training frequency (days)________
Training experience (months)___________
Hazards of physical training ☐ Yes ☐ No
Lasting more than 15 days ☐ Yes ☐ No

QUESTIONNAIRE

1) How many hours do you work and/or study daily?
☐ no work/study (0) ☐ 1 - 4 hours (1) ☐ 4 – 8 hours (2)
☐ 8 – 12 hours (3) ☐ > 12 hours (4)

2) Does your work and/or study require physical and/or intellectual effort?
☐ physical (4) ☐ intellectual (0)

3) Is your exercise session impaired when you work/study hard?
☐ Yes (4) ☐ No (0)

4) Do you work/study tired?
☐ Never (0) ☐ a little (1) ☐ moderately (2) ☐ quite (3) extremely (4)

5) After work and/or study, are you tired?
☐ Never (0) ☐ a little (1) ☐ moderately (2) ☐ quite (3) extremely (4)

6) Is your training/lesson monotonous, boring, or repetitive?
☐ Never (0) ☐ a little (1) ☐ moderately (2) ☐ quite (3) extremely (4)

7) Do you train/work tired?
☐ Never (0) ☐ a little (1) ☐ moderately (2) ☐ quite (3) extremely (4)

8) Do you usually train/work motivated?
☐ Never (0) ☐ a little (1) ☐ moderately (2) ☐ quite (3) extremely (4)

9) How do you rate your workout? (check only one alternative)

6
7 very, very light □
8
9
10 very light □
11
12 slightly light □
13
14 a little heavy □
15
16 heavy □
17
18 very heavy □
19
20 very, very heavy □

10) How do you evaluate your physical performance? (check only one alternative)

6
7 very, very poor performance □
8
9
10 very poor performance □
11
12 poor performance □
13
14 regular performance □
15
16 good performance □
17
18 very good performance □
19
20 very, very good performance □

11) How do you evaluate your physical recovery after the training sessions? (check only one alternative)

6
7 very, very poor recovery □
8
9
10 very poor recovery □
11
12 poor recovery □
13
14 reasonable recovery □
15

16 good recovery □
17
18 very good recovery □
19
20 very, very good recovery □

12) How many times do you rest (do not train/work) weekly?
□ 4 times (0) □ 3 times (1) □ 2 times (2) □ 1 time (3) □ none (4)

13) How many months a year do you do not training/work?
□ none (4) □ < 1 month (3) □ ≥ 1 month (2)

14) Do you usually do steam room, whirlpools, massage, relaxation techniques or therapy?
□ Yes (0) □ No (4)

15) Soon after the training session/work you do some light exercise or stretching?
□ Yes (0) □ No (4)

16) Do you feel physical fatigue during or after training/class/work?
□ Never (0) □ a little (1) □ moderately (2) □ quite (3) extremely (4)

17) Have you felt a lack of energy or desire during the training/work or during the day?
□ Never (0) □ a little (1) □ moderately (2) □ quite (3) extremely (4)

18) Do you consider yourself a person with high motivation to train/work?
□ Never (0) □ a little (1) □ moderately (2) □ quite (3) extremely (4)

19) Do you mind if you do not train/work?
□ Never (0) □ a little (1) □ moderately (2) □ quite (3) extremely (4)

20) Do you consider yourself to be competitive with yourself?
□ Never (0) □ a little (1) □ moderately (2) □ quite (3) extremely (4)

21) How many meals do you usually have per day?
□ 1 □ 2 □ 3 □ 4 □ ≥5

22) How much time after the training/work do you eat?
□ ≤ 2 hours □ 2-3 hours □ 3 - 4 hours □ > 4 hours

23) Do you usually eat bread, pasta, potatoes, cassava or fruits?
□ Never (0) □ a little (1) □ moderately (2) □ quite (3) extremely (4)

24) Do you usually eat chicken, meat, fish, eggs, milk, yogurt or cheese?
□ Never (0) □ a little (1) □ moderately (2) □ quite (3) extremely (4)

25) Do you usually eat vegetables and veggies?
☐ Never (0) ☐ a little (1) ☐ moderately (2) ☐ quite (3) extremely (4)

26) How many glasses of water do you drink daily?
☐ 2-4 ☐ 4-6 ☐ 6-8 ☐ >8

27) How many glasses of water do you drink up to two hours after training?
☐ 1 ☐ 2 ☐ 3 ☐ ≥4

28) How many glasses of water do you drink during your workout?
☐ 1 ☐ 2 ☐ 3 ☐ ≥4

29) Do you take dietary supplements to improve your performance?
☐ Yes ☐ No

30) Are your external obligations and problems currently imapirment your training/work?
☐ No
☐ Yes: Which? ☐ Educational problems. ☐ Professional problems. ☐ Financial problems.
☐ Relationship problems ☐ Losses. ☐ Self esteem

31) How often does this occur? ☐ Never (0) ☐ a little (1) ☐ moderately (2) ☐ quite (3) extremely (4)

32) Did you have any type of injury or illness related to physical exercise? If yes, which are?
☐ Yes____________________________ ☐ No

33) How many hours do you sleep at night?
☐ <4 hours (4) ☐ 4 - 5 hours (3) ☐ 5 - 6 hours (2) ☐ 6 - 7 hours (1) ☐ >7 hours (0)

34) Do you usually have a peaceful sleep?
☐ Never (0) ☐ a little (1) ☐ moderately (2) ☐ quite (3) extremely (4)

35) Do you have trouble to sleeping?
☐ Never (0) ☐ a little (1) ☐ moderately (2) ☐ quite (3) extremely (4)

36) Do you wake up often at night?
☐ Never (0) ☐ a little (1) ☐ moderately (2) ☐ quite (3) extremely (4)

37) Do you usually have nightmares?
☐ Never (0) ☐ a little (1) ☐ moderately (2) ☐ quite (3) extremely (4)

38) Did you have changes in your appetite lately?
☐ Never (0) ☐ a little (1) ☐ moderately (2) ☐ quite (3) extremely (4)

39) Have you been losing weight lately without a diet/regimen?

□ Never (0) □ a little (1) □ moderately (2) □ quite (3) extremely (4)

40) Have you noticed an increase in the amount of sweat lately?

□ Never (0) □ a little (1) □ moderately (2) □ quite (3) extremely (4)

41) Did you have many colds lately?

□ Never (0) □ a little (1) □ moderately (2) □ quite (3) extremely (4)

42) Have you been experiencing many colds for only 1 day lately?

□ Never (0) □ a little (1) □ moderately (2) □ quite (3) extremely (4)

Universidade Federal de Goiás
Faculdade de Educação Física e Dança
Setor de Fisiologia Humana e do Exercício

PROFILE OF MOOD STATES

Directions: Describe HOW YOU FEEL RIGHT NOW by circling the most appropriate number after each of the words listed below:

FEELING	Not al all	A little	Moderate	Quite a bit	Extremally
1. Friendly	1	2	3	4	5
2. Tense	1	2	3	4	5
3. Angry	1	2	3	4	5
4. Worn Out	1	2	3	4	5
5. Unhappy	1	2	3	4	5
6. Clear-headed	1	2	3	4	5
7. Lively	1	2	3	4	5
8. Confused	1	2	3	4	5
9. Sorry for things done	1	2	3	4	5
10. Shaky	1	2	3	4	5
11. Listless	1	2	3	4	5
12. Peeved	1	2	3	4	5
13. Considerate	1	2	3	4	5
14. Sad	1	2	3	4	5
15. Active	1	2	3	4	5
16. On edge	1	2	3	4	5
17. Grouchy	1	2	3	4	5
18. Blue	1	2	3	4	5
19. Energetic	1	2	3	4	5
20. Panicky	1	2	3	4	5
21. Hopeless	1	2	3	4	5
22. Relaxed	1	2	3	4	5
23. Unworthy	1	2	3	4	5
24. Spiteful	1	2	3	4	5
25. Sympathetic	1	2	3	4	5
26. Uneasy	1	2	3	4	5
27. Restless	1	2	3	4	5
28. Unable to	1	2	3	4	5
29. Fatigued	1	2	3	4	5
30. Helpful	1	2	3	4	5
31. Annoyed	1	2	3	4	5
32. Discouraged	1	2	3	4	5
33. Resentful	1	2	3	4	5
34. Nervous	1	2	3	4	5
35. Lonely	1	2	3	4	5
36. Miserable	1	2	3	4	5
37. Muddled	1	2	3	4	5
38. Cheerful	1	2	3	4	5
39. Bitter	1	2	3	4	5
40. Exhausted	1	2	3	4	5

41. Anxious	1	2	3	4	5
42. Ready to fight	1	2	3	4	5
43. Good-natured	1	2	3	4	5
44. Gloomy	1	2	3	4	5
45. Desperate	1	2	3	4	5
46. Sluggish	1	2	3	4	5
47. Rebellious	1	2	3	4	5
48. Helpless	1	2	3	4	5
49. Weary	1	2	3	4	5
50. Bewildered	1	2	3	4	5
51. Alert	1	2	3	4	5
52. Deceived	1	2	3	4	5
53. Furious	1	2	3	4	5
54. Effacious	1	2	3	4	5
55. Trusting	1	2	3	4	5
56. Full of pep	1	2	3	4	5
57. Bad-tempered	1	2	3	4	5
58. Worthless	1	2	3	4	5
59. Forgetful	1	2	3	4	5
60. Carefree	1	2	3	4	5
61. Terrified	1	2	3	4	5
62. Guilty	1	2	3	4	5
63. Vigorous	1	2	3	4	5
64. Uncertain about things	1	2	3	4	5
65. Bushed	1	2	3	4	5
